# Addiction Treatment and Stable Housing among a Cohort of Injection Drug Users

**DOI:** 10.1371/journal.pone.0011697

**Published:** 2010-07-21

**Authors:** Anita Palepu, Brandon D. L. Marshall, Calvin Lai, Evan Wood, Thomas Kerr

**Affiliations:** 1 Centre for Health Evaluation and Outcome Sciences, St. Paul's Hospital, Vancouver, Canada; 2 Division of General Internal Medicine, Department of Medicine, University of British Columbia, Vancouver, Canada; 3 British Columbia Centre for Excellence in HIV/AIDS, Vancouver, Canada; 4 School of Population and Public Health, University of British Columbia, Vancouver, Canada; 5 Division of AIDS, Department of Medicine, University of British Columbia, Vancouver, Canada; RAND Corporation, United States of America

## Abstract

**Background:**

Unstable housing and homelessness is prevalent among injection drug users (IDU). We sought to examine whether accessing addiction treatment was associated with attaining stable housing in a prospective cohort of IDU in Vancouver, Canada.

**Methods:**

We used data collected via the Vancouver Injection Drug User Study (VIDUS) between December 2005 and April 2010. Attaining stable housing was defined as two consecutive “stable housing” designations (i.e., living in an apartment or house) during the follow-up period. We assessed exposure to addiction treatment in the interview prior to the attainment of stable housing among participants who were homeless or living in single room occupancy (SRO) hotels at baseline. Bivariate and multivariate associations between the baseline and time-updated characteristics and attaining stable housing were examined using Cox proportional hazard regression models.

**Principal Findings:**

Of the 992 IDU eligible for this analysis, 495 (49.9%) reported being homeless, 497 (50.1%) resided in SRO hotels, and 380 (38.3%) were enrolled in addiction treatment at the baseline interview. Only 211 (21.3%) attained stable housing during the follow-up period and of this group, 69 (32.7%) had addiction treatment exposure prior to achieving stable housing. Addiction treatment was inversely associated with attaining stable housing in a multivariate model (adjusted hazard ratio [AHR]  = 0.71; 95% CI: 0.52–0.96). Being in a partnered relationship was positively associated with the primary outcome (AHR  = 1.39; 95% CI: 1.02–1.88). Receipt of income assistance (AHR  = 0.65; 95% CI: 0.44–0.96), daily crack use (AHR  = 0.69; 95% CI: 0.51–0.93) and daily heroin use (AHR  = 0.63; 95% CI: 0.43–0.92) were negatively associated with attaining stable housing.

**Conclusions:**

Exposure to addiction treatment in our study was negatively associated with attaining stable housing and may have represented a marker of instability among this sample of IDU. Efforts to stably house this vulnerable group may be occurring in contexts outside of addiction treatment.

## Introduction

Addiction and homelessness are often co-occurring conditions. Addiction is characterized by the persistent use of alcohol or drugs despite negative consequences to the one's health and the loss of social functioning related to the substance use. Untreated, addiction can result in significant morbidity and mortality [1], [2]. Homelessness is also an independent risk factor for morbidity and mortality [3]–[7] and both addiction and homelessness are associated with significant service utilization and costs to the health, criminal justice and social welfare systems [8]–[13]. The underlying reasons for homelessness are complex, although previous studies have shown that substance use (illicit drugs and alcohol) is prevalent among persons who are homeless [14]–[16], with substance use potentially being a cause or consequence of homelessness.

In order to mitigate the harms associated with a lack of housing, numerous trials have been conducted to examine the impact of housing interventions such as “Housing First” approaches on the residential trajectory of chronically homeless persons with severe and persistent mental illness. The proportion of study participants in the Housing First studies who had severe substance use problems was relatively low [17], potentially limiting its generalizability to this group. Recently published studies of Housing First have found favourable outcomes among chronically homeless alcoholics in Seattle; however, there was no mention of illicit drug use in the sample [18]. A study of homeless persons in Chicago with chronic medical conditions who used the medical services frequently also reported a reduction in medical service utilization. Of their sample, 60% reported using illicit drugs in the previous 30 days but no further details regarding type or frequency of drug use were provided [19]. Thus, these findings may not be generalizable to active drug users.

In contrast, linear approach programs [20], common in the United States, are focused on achieving abstinence and require persons who are homeless and have substance abuse problems to engage in intensive addiction treatment programs as a prerequisite to maintaining temporary housing. One linear approach program has been extensively studied over the past 12 years in Birmingham, Alabama and uses abstinence contingent housing among homeless persons with cocaine dependence [21]–[24]. A meta-analysis of the four randomized controlled trials found that drug abstinence was higher in the abstinent contingent housing arm compared to the arm that offered only day treatment (58% vs. 26%) at six months [25]. Interestingly, a small retrospective study that compared homeless patients receiving office-based buprenorphine treatment to housed patients found that the homeless patients had similar outcomes with respect to illicit drug use, treatment failure and use of addiction treatment despite having higher social instability, greater comorbidities, and more chronic drug use compared to the housed individuals in this study. Furthermore, 36% of the homeless group was housed at twelve months [26]. The focus of addiction treatment is generally to reduce illicit drug use and improve social, vocational and interpersonal functioning, which would include attaining stable housing for homeless individuals.

Illicit drug use is prevalent in the Downtown Eastside neighbourhood of Vancouver, Canada and the proportion of drug users who are also using crack has been increasing [27]–[29]. Addiction treatment, including methadone maintenance therapy, addiction counseling and self-help groups are available through various community health centers, which also provide primary care health services throughout Vancouver and are covered by universal health care [30]. We have been following a cohort of injection drug users and have detailed longitudinal data on their drug use, addiction treatment history, and housing status. We hypothesized that addiction treatment exposure in IDUs would increase their probability of attaining stable housing due to reduced illicit drug use, improved social functioning, and increased overall stability. In this manuscript, we describe the proportion of participants who accessed the various available addiction treatment modalities, and examine the association between enrolment in addiction treatment and the attainment of stable housing over time. Given that there are few observational data on the relationship between addiction treatment and housing status among injection drug users, the study findings may inform the design and implementation of evidence-based addiction treatment and supportive housing interventions for this vulnerable population.

## Methods

### Ethics Statement

The University of British Columbia/Providence Health Care Research Ethics Board has approved this study.

### Measures

The Vancouver Injection Drug Users Study (VIDUS) is an ongoing prospective cohort study of injection drug using individuals recruited through self-referral and street outreach from Vancouver's Downtown Eastside. The study has been described in detail previously [31], [32]. Briefly, persons were eligible to participate in VIDUS if they had injected illicit drugs at least once in the previous six months, resided in the Greater Vancouver region at time of enrolment, and provided written informed consent. At baseline and semi-annually, subjects complete an interviewer-administered questionnaire. The questionnaire elicits demographic data as well as information regarding drug use, HIV risk behaviours, housing status, mental health diagnoses, addiction treatment and hospitalization. Participants receive $20 (CDN) for each study visit.

For the purpose of this analysis, we included participants who completed a baseline questionnaire between December 2005 - May 2006 and at least two of eight subsequent follow-up questionnaires ending in April 2010. We chose this recent period to reflect the current context of addiction treatment, drug use and housing status in the community and among cohort participants. All participants who reported currently living in stable housing at the date of their baseline interview were excluded from this analysis.

The primary outcome was attaining stable housing, defined as self-reported living in an apartment or a house over two consecutive interviews during the follow-up period. Stable housing did not include living in a recovery house or residential treatment centre. Participants who remained homeless or who lived in single room occupancy (SRO) hotels were classified as having unstable housing as these situations have been associated with poor health outcomes [10], [33], [34]. Participants were censored once they achieved the primary outcome or at the end of the follow-up period. We considered current addiction treatment as being engaged in any of the following: recovery house, residential treatment centre, addiction counseling, self-help groups and methadone maintenance treatment. We defined the drug use and alcohol variables as at least daily use of beer, hard liquor, crack cocaine and injection cocaine, heroin, crystal methamphetamine. We defined heavy alcohol use as consuming on average greater than four drinks per day [35]. Participants who were legally married, common-law or had a regular partner were classified as having a “partnered” relationship status. Current employment was defined as being legally employed at the time of the interview. Income assistance was defined as receiving income assistance in the past six months.

### Statistical Analysis

We examined the bivariate and multivariate associations between the baseline and time-updated characteristics and attaining stable housing using Cox proportional hazard regression models. An event was defined as the date of the interview during which the first of two consecutive stable housing designations were reported. Addiction treatment exposure was examined in the interview prior to the interview when the first of two stable housing designations was attained. We fitted a multivariate Cox proportional hazard regression model using a backward elimination procedure for variable selection [36] based on the Akaike information criterion (AIC). In addition, we forced gender, age, Aboriginal ancestry, legal employment status, and education level (at least high school) in the final model. We also examined the interaction between cocaine (crack and injection cocaine) and addiction treatment given that this was the most common drug type used on a daily basis. The proportional hazards assumption was evaluated by using log-log survival curves for select baseline variables. The results are reported as adjusted hazard ratios (AHRs) and 95% confidence intervals (CI). All statistical analyses were performed using SAS software version 9.1.3 (SAS, Cary, NC), and all reported *p*-values are two-sided. We also conducted a number of sub-analyses to explore the relationship between various addiction treatment modalities and stable housing attainment, including those who were not on methadone maintenance at baseline but started this treatment during the follow up period (incident methadone maintenance).

## Results

In total, 992 IDU participants eligible for this analysis. The median age of the cohort was 42.2 years (IQR: 35.9–47.1), just over one-third (N = 327) were female, 32.1% (N = 331) self-identified as of Aboriginal ancestry (i.e., First Nations, Inuit, or Métis), and 42.8% (N = 425) reported ever being diagnosed with a mental illness. The prevalence of the various mental health diagnoses was: depression (29.3%); anxiety disorder (12.2%); bipolar affective disorder (7.6%); post-traumatic stress disorder (6.1%); attention deficit disorder with or without hyperactivity (5.9%); and schizophrenia (3.0%). At baseline the proportion of the participants using drugs at least daily was substantial: crack cocaine (43.0%); injection cocaine (10.4%); and injection heroin (27.1%).

Of the 992 participants, 211 (21.3%) attained stable housing during the follow-up period for an incidence density of 9.7 (95% CI 8.4–11.0) per 100 person-years. At baseline, 380 (38.3%) were currently engaged in some form of addiction treatment at the baseline interview. During the follow-up period, there were 74 (7.5%) additional participants who reported addiction treatment exposure. Of the 992 participants, 811 (81.8%) reported that they had ever been homeless; 495 (50%) were homeless at baseline and of them 80 (26.4%) obtained stable housing during the follow up period. There were 497 participants who resided in SRO hotels at baseline: during the follow-up period, 366 (73.6%) remained in this form of housing and 131 (26.3%) attained stable housing in an apartment or house. **Table 1** presents the cumulative distribution of the various addiction treatment modalities accessed at baseline and follow-up; methadone maintenance therapy was by far the most prevalent addiction treatment (36.1%) in this cohort.

**Table 1:** Cumulative (baseline and follow-up) utilization of addiction treatment modalities among a prospective cohort of injection drug users (N = 992).

Addiction Treatment Modality	N (%)	Median Duration Days (IQR)
**Recovery House**	18 (1.8)	83 (26–94)
**Treatment Centre**	3 (0.3)	-
**Addiction counsellor**	44 (4.4)	135 (64–730)
**Self-help (AA/NA/CA)**	31 (3.1)	730 (136–3098)
**Methadone maintenance therapy**	358 (36.1)	731 (244–2188)

The baseline characteristics associated with attaining stable housing are presented in **Table 2**. The only factor positively associated with the primary outcome was having current legal employment (hazard ratio [HR]  = 1.61; 95% CI: 1.19–2.16). Factors negatively associated with attaining stable housing were being homeless at baseline (HR  = 0.47; 95% CI: 0.36–0.62), receipt of income assistance (HR  = 0.58; 95% CI: 0.40–0.84), daily crack use (HR  = 0.65; 95% CI: 0.48–0.86), and daily injection heroin use (HR  = 0.62; 95% CI: 0.43–0.89). Current enrolment in addiction treatment was also negatively associated with attaining stable housing in follow-up (HR  = 0.72; 95% CI: 0.54–0.96).

**Table 2:** Prevalence of baseline characteristics and bivariate associations (hazard ratios) between each characteristic and attaining stable housing.

Attain Stable Housing†				
Characteristic	Yes n = 211 n (%)	No n = 781 n (%)	Hazard Ratio (95% CI)	*p -* value
**Median age (IQR)**	44 (38–49)	42 (35–47)	1.01 (1.00–1.03)	0.160
**Female sex**	67 (31.8)	260 (33.3)	0.95 (0.71–1.26)	0.703
**Aboriginal ancestry**	76 (36.0)	255 (32.7)	1.08 (0.82–1.43)	0.577
**≥ High school education**	107 (50.7)	370 (47.4)	1.09 (0.83–1.43)	0.529
**Current legal employment**	63 (29.9)	169 (21.6)	1.61 (1.19–2.16)	0.002
**Partnered relationship**	65 (30.8)	219 (28.0)	1.22 (0.91–1.64)	0.176
**Received income assistance** *	177 (83.9)	697 (89.2)	0.58 (0.40–0.84)	0.004
**Current homelessness**	80 (37.9)	415 (53.4)	0.47 (0.36–0.62)	0.001
**Crack use** ‡ *	68 (32.2)	359 (46.0)	0.65 (0.48–0.86)	0.001
**Greater than 4 drinks/days** *	49 (23.2)	169 (21.6)	1.19 (0.86–1.64)	0.293
**Injection cocaine use** ‡ *	17 (8.1)	86 (11.0)	0.77 (0.47–1.27)	0.312
**Injection heroin use** ‡ *	35 (16.6)	234 (30.0)	0.62 (0.43–0.89)	0.009
**Injection crystal methamphetamine use** ‡ *	6 (2.8)	27 (3.5)	0.91 (0.40–2.06)	0.824
**Current addiction treatment**	69 (32.7)	311 (39.8)	0.72 (0.54–0.96)	0.026
**Ever diagnosed with mental illness**	88 (41.7)	337 (43.1)	0.92 (0.70–1.21)	0.537
**Current mental health treatment**	44 (20.9)	134 (17.2)	1.10 (0.78–1.54)	0.586
**Hospitalization for medical condition** *	43 (20.4)	151 (19.3)	1.12 (0.80–1.57)	0.523

Notes:

† stable housing refers to living in an apartment or house over two consecutive follow-ups;

‡ at least daily use;

* refers to activities in the past 6 months; time-dependent variables in the above table are time-updated in the bivariate Cox proportional hazards regressions.

In the multivariable Cox proportional hazards regression model shown in **Table 3**, addiction treatment exposure in the interview prior to attaining stable housing was negatively associated with our primary outcome (adjusted hazard ratio [AHR]  = 0.70; 95% CI: 0.52–0.96). Being in a partnered relationship (AHR  = 1.39; 95%CI: 1.02–1.88) remained independently associated with attaining stable housing. Daily crack use (AHR  = 0.69; 95% CI: 0.51–0.93), daily injection heroin use (AHR  = 0.63; 95% CI: 0.43–0.92) and receipt of income assistance (AHR  = 0.65; 95% CI: 0.44–0.96) remained negatively associated with stable housing attainment. We found no interaction between cocaine use and addiction treatment on the likelihood of attaining stable housing (p = 0.38 for crack and p = 0.95 for injection cocaine). When we examined the bivariate associations of the various addiction treatment modalities, each were negatively although non-significantly associated with stable housing attainment (**Table 4**).

**Table 3:** Cox proportional hazards analysis of factors associated with attaining stable housing (N = 992).

Variable	Adjusted Hazard Ratio (AHR)	95% Confidence Interval (CI)	*p -* value
**Age**			
(per year)	1.01	1.00–1.03	0.165
**Female sex**			
(yes vs. no)	1.14	0.83–1.57	0.428
**Aboriginal ancestry**			
(yes vs. no)	1.01	0.75–1.36	0.963
**≥ High school education**			
(yes vs. no)	1.04	0.79–1.37	0.804
**Current legal employment**			
(yes vs. no)	1.35	0.98–1.86	0.062
**Partnered relationship**			
(yes vs. no)	1.39	1.02–1.88	0.036
**Received income assistance** †			
(yes vs. no)	0.65	0.44–0.96	0.031
**Crack use** †			
(≥ daily vs. < daily)	0.69	0.51–0.93	0.015
**Injection heroin use** †			
(≥ daily vs. < daily)	0.63	0.43–0.92	0.016
**Current addiction treatment**			
(yes vs. no)	0.71	0.52–0.96	0.025

Note:

† refers to activities in the past 6 months; time-dependent variables in the above table are time-updated in the multivariate Cox proportional hazards regression model.

**Table 4:** Bivariate associations (hazard ratios) between addiction treatment modalities and attaining stable housing.

Attain Stable Housing†				
Addiction Treatment Modality (yes versus no)	Yes n = 211 n (%)	No n = 781 n (%)	Hazard Ratio (95% CI)	*p -* value
**Any methadone maintenance**	72 (34.1%)	286 (36.6%)	0.95 (0.72–1.26)	0.724
**Incident methadone maintenance**	48 (22.7%)	192 (24.6%)	0.71 (0.50–1.00)	0.050
**Addiction counselling &/0r self-help**	8 (3.8%)	67 (8.6%)	0.62 (0.39–1.00)	0.050
**Recovery house &/or residential treatment**	4 (1.9%)	17 (2.2%)	0.83 (0.31–2.23)	0.707

Notes:

† stable housing refers to living in an apartment or house over two consecutive follow-ups; the independent variables in the above table are time-updated in the bivariate Cox proportional hazards regressions.

## Discussion

In this prospective cohort study of injection drug users, current enrolment in addiction treatment was negatively associated with attaining stable housing, even after adjustment for potential confounders including relationship status, employment status and drug use. The negative association of addiction treatment prior to attaining stable housing is somewhat unexpected, but may reflect the inadequacy of appropriate treatment exposure to meaningfully impact long-term housing outcomes. These findings also suggest that accessing addiction treatment may be a marker of instability (i.e., periods of extreme vulnerability during which other basic necessities including food and shelter take precedence over obtaining stable housing). Of note, the vast majority of the addiction treatment services offered in Vancouver do not have a formal linkage with permanent housing placement [30]. It may also reflect the reality of the exceedingly low rental vacancy rate in Vancouver [37], as it is currently very challenging for persons with addictions to access independent apartments and houses, especially if they are actively using drugs as observed in our cohort and in other studies [38], [39].

Over one-third of our study participants reported engagement in methadone maintenance therapy at baseline (36.1%, N = 358), which targets opiate addiction, while exposure to other modalities that are not drug specific such as recovery houses, residential treatment, addiction counseling and self-help groups was low in this cohort. In fact, ongoing daily crack use negatively predicted attaining stable housing. This may reflect the many barriers that active drug users face in attempting to obtain or maintain stable housing and the adverse effects of ongoing drug use on efforts to do so. North *et al.* followed 400 homeless persons over two years and also found that ongoing cocaine use was negatively associated with the attainment of stable housing [40]. Unlike heroin addiction, there are no efficacious pharmacologic therapies for cocaine addiction and the accepted approach is cognitive behavioral therapy [41]. Given the prevalence of daily crack and injection drug use in our cohort, the exposure to addiction counselling (4.4%) is very low and it appears that most users are not accessing meaningful cocaine addiction treatment [42]. Marsden et al recently reported the effectiveness of community treatments (pharmacologic and psychosocial) for heroin and crack cocaine addiction in England and found that at six months, there was 37% complete abstinence from heroin and 52% complete abstinence from crack cocaine. They also noted that pharmacological treatment was less effective among users of both heroin and crack cocaine, who comprised 51% of our cohort [43].

The linear treatment intervention tested in the Birmingham model was comprised of abstinent-contingent housing for 6 months with behavioral treatment and employment training for 6–8 hours per day for homeless, cocaine-dependent treatment-seekers. [21]–[24] In the third trial, clients who were assigned abstinent-contingent housing had a higher proportion in stable housing at 6 months (42% of 45) compared to clients who had housing not contingent on abstinence (33% of 54) and participants in treatment who had to find their own accommodation (26% of 39) [23]. This comparison did not reach statistical significance, likely due to the small sample size. In contrast, we found a negative association of addiction treatment and stable housing; unlike these trials we did not have a linked housing intervention. In Birmingham and in other jurisdictions, there is little ability within the housing stock to accommodate persons who are unable to achieve abstinence and although the housing status does improve for many, a substantial proportion are unable to access stable housing, highlighting the need for the integration of addiction treatment services and supportive housing to target persons who are unable to achieve abstinence [44].

In Kertesz' review of the strengths and weaknesses of linear and Housing First approaches, he notes that they target different primary problems, namely housing retention vs. addiction and the achievement of abstinence [38]. Studies of these two approaches have recruited different sub-populations of the chronically homeless. Most Housing First trials included persons with severe and persistent mental illness [45]–[47], with the exception of the Chicago trial of the homeless with chronic medical conditions accessing the emergency department [19] and the Seattle study of severe alcoholics [18]. The economic benefits found in these latter studies were related to the inclusion of chronically homeless persons who were high users of health and other public services and this may not be generalizable to all homeless persons [48]. The linear approach trials included homeless cocaine-dependent persons seeking addiction treatment with the intervention goal being abstinence [21]–[24]. The transition to market housing and long-term housing retention can be challenging as not all clients were able to remain abstinent [38].

Interestingly, we observed that a high proportion of our study participants who did not have prior addiction treatment exposure achieved stable housing over follow-up, suggesting that this group was more capable of accessing housing services that may have helped achieve this outcome and are quite separate from addiction treatment. This highlights the need for supportive housing with integrated addiction treatment services for the chronically homeless that are seeking treatment, given that housing stability is an important functional outcome.

Our study had several limitations. This was an observational study and the addiction treatment reflected what cohort participants accessed during the study period and likely represents usual care for persons who are active illicit drug users in our setting. As a result, our definition of addiction treatment was broad and may not have been stringent enough to provide sufficient exposure and duration to impact drug use and thereby improve the participant's housing status. However, our study did consider addiction treatment exposure during a six-month period, and other studies have reported positive outcomes following the provision of addiction treatment over six months [43]. The negative association may also reflect the selection of the most heavy drug users who accessed addiction treatment and are less likely to be housed because of the intensity of their addiction. Like all observational studies, residual confounding may be present in this instance. However, it should be noted that our analyses included adjustment for a range of potential confounders, including intensity of drug and alcohol use. Finally, the study population was a non-random sample and our findings may have limited generalizability to other injection drug user populations.

In summary, we found that injection drug users who accessed addiction treatment services were less likely to attain stable housing compared to those who did not. In our study, addiction treatment exposure may have been a marker of life instability. Frequent (i.e., daily) drug use was prevalent among our study participants, particularly crack cocaine and injection heroin, and this was negatively associated with attaining stable housing. The exposure to addiction treatment services may not have been potent enough to reduce drug use sufficiently for participants to be able to access stable housing. Future studies should evaluate the formal linkage of addiction treatment and supportive housing services as a strategy to improve the health and housing status of this vulnerable population, as addiction treatment in our study did not positively impact the attainment of stable housing.
